# Missense variants in the conserved transmembrane M2 protein domain of *KCNJ13* associated with retinovascular changes in humans and zebrafish

**DOI:** 10.1016/j.exer.2019.107852

**Published:** 2019-12

**Authors:** Maria Toms, Adam M. Dubis, Wei Sing Lim, Andrew R. Webster, Michael B. Gorin, Mariya Moosajee

**Affiliations:** a UCL Institute of Ophthalmology, London, UK; b Moorfields Eye Hospital NHS Foundation Trust, London, UK; c Department of Ophthalmology, Jules Stein Eye Institute, David Geffen School of Medicine, UCLA Los Angeles, USA

## Abstract

Mutations in *KCNJ13* are associated with two retinal disorders; Leber congenital amaurosis (LCA) and snowflake vitreoretinal degeneration (SVD). We describe a novel fibrovascular proliferation in the retina of two affected members of a *KCNJ13*-related LCA family with a homozygous c.458C > T, p.(Thr153Ile) missense mutation. Optical coherence tomography retinal imaging of the *kcnj13* mutant zebrafish (*obelix*^*td15*^ c.502T > C, p.[Phe168Leu]) revealed a late onset retinal degeneration at 12 months, with retinal thinning and associated retinovascular changes, including increased vessel calibre and vitreous deposits. Both human and zebrafish variants are missense and located within the conserved transmembrane M2 protein domain, suggesting that disruption of this region may contribute to retinovascular changes as an additional feature to the previously described LCA phenotype. Close monitoring of other patients with similar mutations may be required to minimise the ensuing retinal damage.

## Highlights

• Patients with *KCNJ13* c.458C>T, p.(Thr153Ile) mutations were examined for retinovascular abnormalities.
• A novel fibrovascular proliferation was observed in the retina of two siblings with *KCNJ13*-Leber congenital amaurosis.
• *kcnj13* mutant zebrafish (*obelix*^*td15*^) showed a retinal degeneration with associated retinovascular changes.
• Both human and zebrafish variants are missense and located within the transmembrane M2 protein domain.
• This retinovascular complication should be considered within the clinical spectrum of *KCNJ13*-related retinal disease.

## 1 Introduction

The *KCNJ13* gene (potassium voltage-gated channel subfamily J member 13) encodes Kir7.1, a member of the inwardly-rectifying potassium channel family (Krapivinsky et al., 1998; Partiseti et al., 1998; Doring et al., 1998). The Kir7.1 protein consists of a pore domain flanked by two transmembrane regions and functions as a homotetramer. The channel is expressed at the plasma membrane of a variety of ion-transporting epithelia, including the retinal pigment epithelium (RPE) (Kusaka et al., 2001; Hejtmancik et al., 2008; Yang et al., 2008). In the RPE, Kir7.1 is localized to the apical membrane at the interface with the photoreceptor outer segments, where it facilitates potassium ion (K^+^) efflux to the subretinal space (Yang et al., 2003) and is involved in controlling fluid flow across the blood–retina barrier (Hughes and Takahira, 1996; Doring et al., 1998). Kir7.1 shows co-localization with the Na^+^/K^+^ pump, suggesting that it is involved in K^+^ recycling required to keep up with high rates of epithelial ion transport (Nakamura et al., 1999).

Mutations in *KCNJ13* have been linked with two ocular disorders; (i) autosomal recessive Leber congenital amaurosis (LCA, MIM #614186) (Sergouniotis et al., 2011; Pattnaik et al., 2015; Perez-Roustit et al., 2017), and (ii) autosomal dominant snowflake vitreoretinal degeneration (SVD, MIM #193230) (Hejtmancik et al., 2008). LCA is a severe early onset retinal dystrophy with RPE and photoreceptor loss causing blindness from birth (Kumaran et al., 2017). It is characterized by sensory nystagmus, amaurotic pupils and absent electrical signals on an electroretinogram. Retinal features including macular atrophy, pigment deposits and vessel attenuation have been reported in LCA patients with *KCNJ13* mutations (Sergouniotis et al., 2011; Pattnaik et al., 2015; Perez-Roustit et al., 2017). SVD is a disorder characterized by a fibrillar vitreous degeneration, peripheral retinal abnormalities including small inner retinal crystalline-like deposits resembling snowflakes and chorioretinal atrophy, optic nerve head dysmorphism, early-onset cataracts and corneal guttae (Hejtmancik et al., 2008). The visual acuity is normal and retinal function testing reveals only mild abnormalities.

Kir7.1 is well conserved and defects in this channel have been found to cause retinal disease in both mice (Zhong et al., 2015; Roman et al., 2018) and zebrafish (Toms et al., 2019). The *obelix* (*obe*^*td15*^) zebrafish mutant, generated through ENU mutagenesis, harbors a missense mutation (c.502T > C, p.Phe168Leu) in *kcnj13*, which affects the transmembrane region abolishing K^+^ conductance by disrupting K^+^ permeation through the channel (Iwashita et al., 2006). Previously, we have described the retinal disease in this zebrafish model which corresponds to the changes seen in patients with *KCNJ13*-related LCA (Toms et al., 2019). Here, we describe retinovascular abnormalities not reported previously in a human family with c.458C > T, p.(Thr153Ile) *KCNJ13* mutations with corresponding retinovascular defects in the *obe*^*td15*^ zebrafish mutant suggesting an additional clinical feature of this condition.

## 2 Materials and methods

### 2.1 Patient evaluation

The study was approved by the local research ethics committee, and all investigations were conducted in accordance with the principles of the Declaration of Helsinki; informed consent was obtained from all participating individuals. Molecular genetic testing on genomic DNA extracted from blood using an LCA next-generation sequencing (NGS) panel including *KCNJ13* [MIM #603208], with Sanger sequencing to confirm the mutations. Ophthalmic evaluation included slit lamp examination, fundus examination and fundus fluorescein angiogram (FFA) with Optos imaging, as part of routine clinical care.

### 2.2 Zebrafish husbandry

Wild-type AB and *obe*^*td15*^ zebrafish were generated by natural pair-wise matings of genotyped homozygous or heterozygous fish and raised at 28.5 °C on a 14 h light/10 h dark cycle in the UCL zebrafish facility. Zebrafish were maintained according to local UCL and UK Home Office regulations for the care and use of laboratory animals under the Animals Scientific Procedures Act at the UCL Institute of Ophthalmology animal facility. UCL Animal Welfare and Ethical Review Body approved all procedures for experimental protocols, in addition to the UK Home Office (License no. PPL PC916FDE7). All zebrafish experimentation followed the ARRIVE guidelines for animal research.

### 2.3 Optical coherence tomography (OCT) zebrafish imaging

Wild-type and *obe*^*td15*^ retinas were scanned using the Bioptigen Envisu R2200 Spectral Domain Ophthalmic Imaging System (Bioptigen, Inc.) as described (Toms et al., 2017). The approximate same region of dorsal retina was imaged in each zebrafish using a 1 × 1 mm perimeter scan with 400 A-scans per B-scan with 400 total B-scans. To assess anatomical vessel differences, en face slabs were generated using custom matlab software (Mathworks, Inc). These slabs were defined at 10 μm below the inner limiting membrane to 40 μm above. This provided a map of the retinal vasculature. Measurements of vessel width were extracted using longitudinal reflectivity profiles (LRP) (Huang et al., 1998). Distance from the edge of the optic nerve was also calculated. If the optic nerve was not visible in the scan, distance from the optic nerve was calculated using the angles of the vessels. Measurements were taken from 3 to 7 locations per fish. The slopes of vessel width by location were compared between the wild-type and mutant zebrafish. A multi-linear model for mixed effect testing was used for statistical analysis, which was performed using JMP13 (SAS Institute Inc).

## 3 Results

### 3.1 Retinal vasculature changes in patients with *KCNJ1*3-LCA

**Fig. 1:**
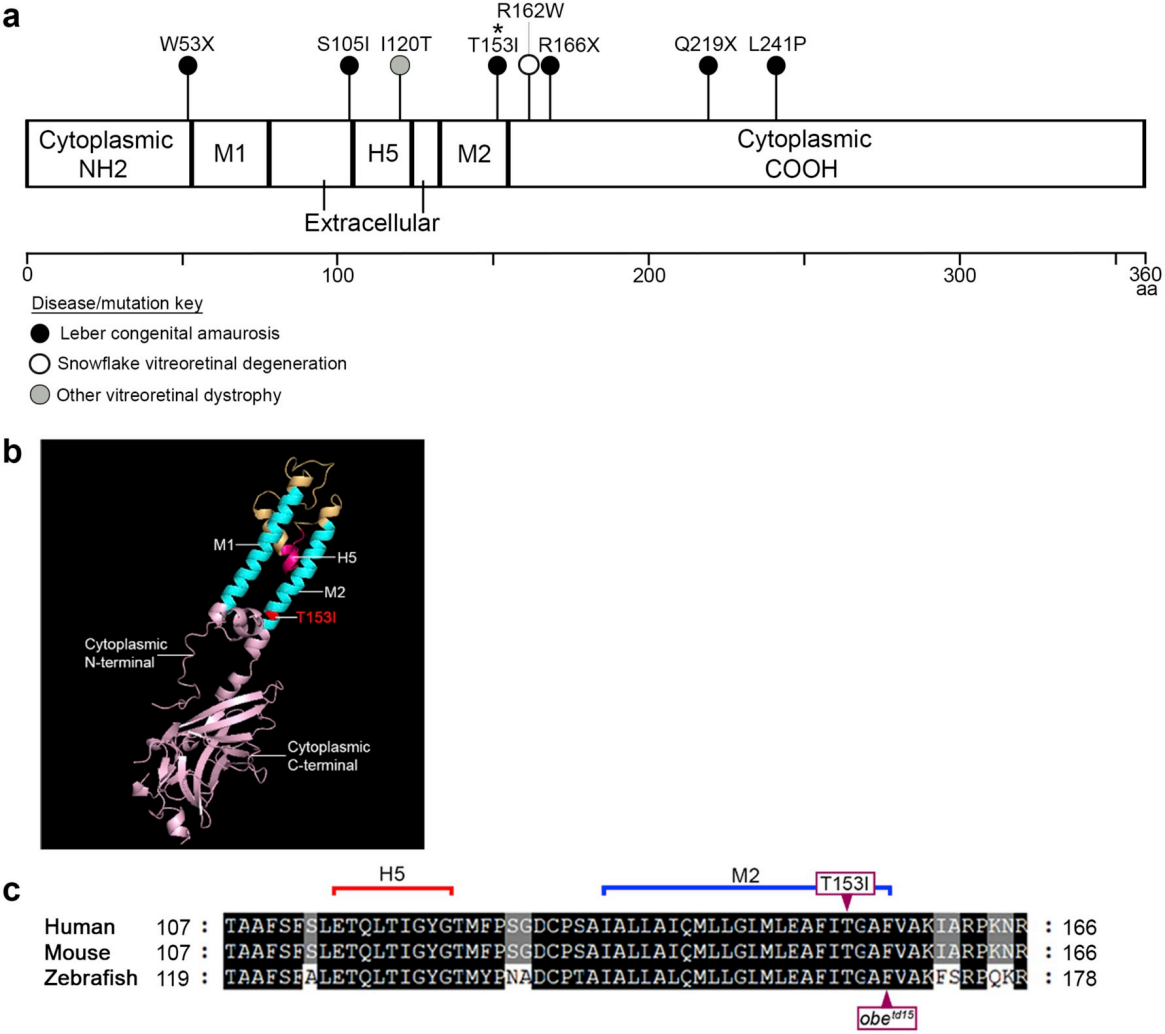
Location of reported *KCNJ13* mutations on the Kir7.1 protein. (a) Schematic of the linear structure of Kir7.1 shows two transmembrane α helices (M1 and M2) with cytoplasmic NH2 and COOH termini, separated by an extracellular pore-forming loop that acts as a selectivity filter (H5). The location of published mutations is indicated and color-coded according to their associated disease. The missense mutation (p.Thr153Ile [T153I]) identified in families A and B in this study is highlighted with *. (b) Human Kir7.1 monomer model generated using Phyre2; the crystal structure of Kir3.2 was used as a template. The T153I mutation is highlighted in red. (c) Alignment of the human, mouse and zebrafish Kir7.1 protein sequences demonstrates the close proximity of the *obelix*^*td15*^ (*obe*^*td15*^) zebrafish missense change (p.Phe168Leu) to the patient T153I mutation within the fully conserved M2 domain. (For interpretation of the references to color in this figure legend, the reader is referred to the Web version of this article.)

Two unreported and unrelated LCA families, diagnosed in infancy, were found to carry the same homozygous missense mutation c.458C > T, p.(Thr153Ile) in *KCNJ13* (Table 1, Fig. 1). This variant was previously identified in the supplementary data from Lee et al. (2014). This change is located in exon 2 of the gene within the coding region for the M2 transmembrane domain of Kir7.1. The c.458C > T, p.(Thr153Ile) variant was found to have a CADD score of 26.8 (damaging) and PolyPhen2 score of 1 (probably damaging). It is also predicted to be damaging/disease-causing by SIFT and MutationTaster.

**Table 1:** **Leber congenital amaurosis (LCA) and snowflake vitreoretinal degeneration (SVD) patients with *KCNJ13* mutations examined in this study and previous publications.** RVA, right visual acuity; LVA, left visual acuity; FFA, fundus fluorescein angiogram; CF, count fingers; NPL, no light perception; PRP, pan-retinal photocoagulation; logMAR, logarithm of the minimal angle of resolution. All patients had LCA except for where marked *SVD family.

Patient	Age	Mutation	RVA	LVA	FFA
A-1	20	c.458C > T, p.Thr153Ile	CF	NPL	Retinal neovascularization, diffuse severe leakage (early leakage primarily associated with areas of prior fibrosis along arcades and disc), had extensive PRP.
A-2	18	c.458C > T, p.Thr153Ile	CF	1.2 logMAR	Right vitreous haemorrhage with diffuse severe leakage.
B-1	13	c.458C > T, p.Thr153Ile	1.3 logMAR	1.3 logMAR	Very mild leakage from the disc in both eyes but with no neovascularization elsewhere and no peripheral vessel leakage.
B-2	10	c.458C > T, p.Thr153Ile	CF	CF	No evidence of blood vessel leakage or neovascularization elsewhere.
Sergouniotis et al. (2011)					
Patient A-2	34	c.496C > T p.Arg166*	2.0 logMAR	2.0 logMAR	–
Patient A-3	32	c.496C > T p.Arg166*	1.78 logMAR	1.48 logMAR	–
Patient B-3	33	c.722T > C p.Leu241Pro	1.45 logMAR	1.45 logMAR	–
Khan et al. (2015)					
Case 1	12	c.359T > C p.Ile120Thr	Hand motion	0.7 logMAR	–
Case 2	33	c.359T > C p.Ile120Thr	1.3 logMAR	1.3 logMAR	–
Pattnaik et al. (2015)	10	c.158G > A p.Trp53*	2.0 logMAR	CF	–
Perez-Roustit et al. (2017)	31	c.314G.T p.Ser105Ile, c.655C.T p.Gln219*	1.0 logMAR	1.3 logMAR	–
* Hejtmancik et al. (2008) Lee et al. (2003) 13 family members with SVD	12–85	c.484C > T p.Arg162Trp	See Table 1 in (Lee et al., 2003)		–

**Fig. 2:**
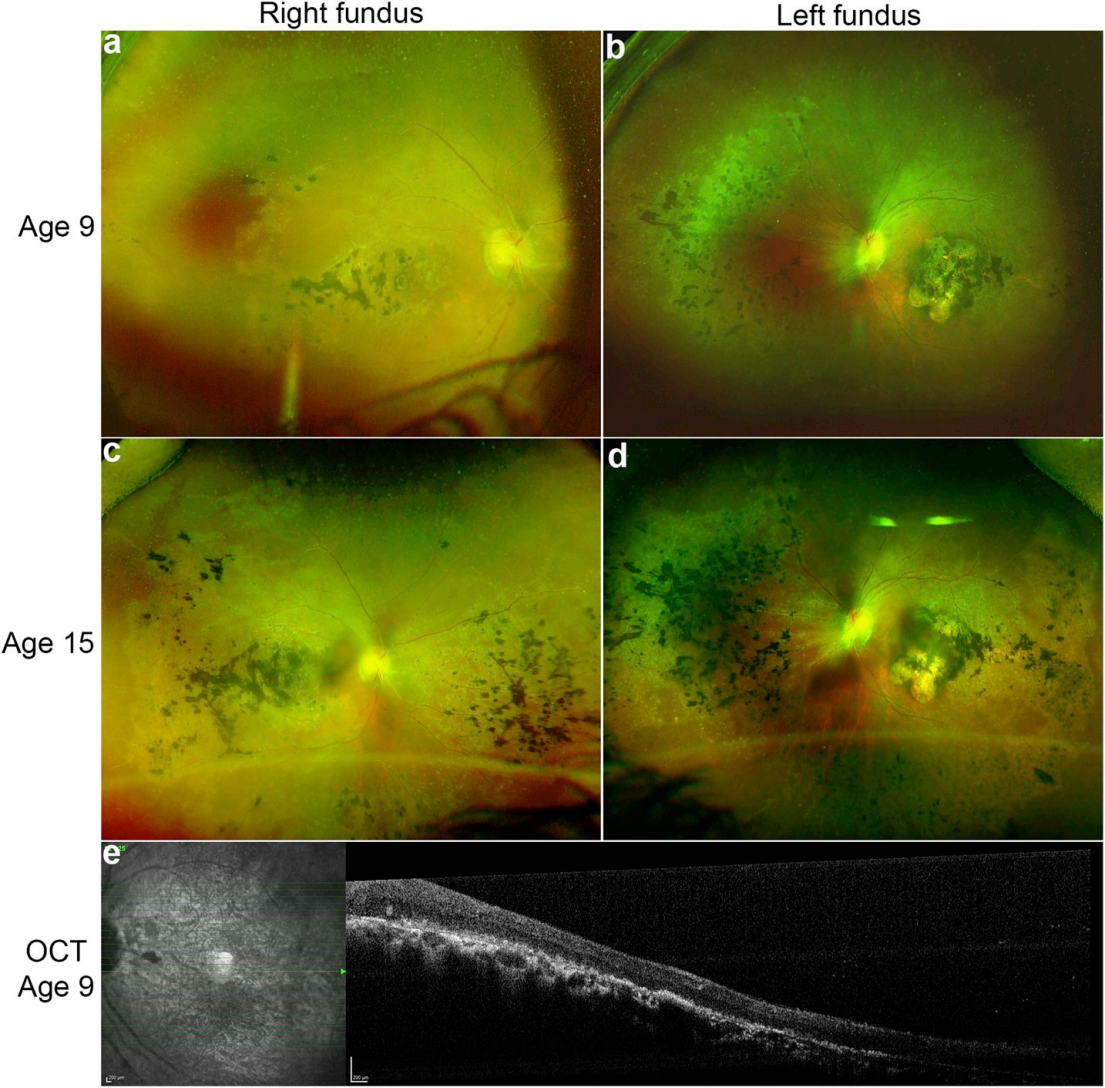
Early clinical features of the *KCNJ13* retinopathy. Ultra-wide field fundus imaging with Optos (Dunfermline, Scotland) of the right and left eye from patient A-2 with a missense mutation (p.Thr153Ile) in *KCNJ13* at age 9 (a, b) and at age 15 (c, d). It shows early stage retinal vessel attenuation and progressive retinal pigmentation in a nummular pattern. At age 15, signs of preretinal fibrosis can be seen over the optic disc extending across the arcade. OCT of the left eye at age 9 (e), prior to the onset of vitreous haemorrhage, reveals the presence of retinal lamination but with foveal hypoplasia; this was also seen in patient A-1.

Both families were investigated for retinovascular changes. The affected siblings in family A developed retinal neovascularization with diffuse severe leakage and vitreous haemorrhages at age 17 on a background of characteristic *KCNJ1*3-LCA (with areas of nummular pigment at the level of the RPE, especially over the posterior pole, macular atrophy and optic disc pallor with retinal vessel attenuation). The initial appearances of the retinas in the two brothers looked fairly typical of LCA with extensive RPE changes in both the macula and retinal peripheries with progressive pigment migration and vessel attenuation (Fig. 2a–d). However, prior to the development of the vitreous haemorrhage, there was preretinal fibrosis over the disc and along the arcade. Unlike typical foci of neovascularization, there was diffuse leakage from the retinal vessels and a diffuse vaso-proliferative response. OCT imaging showed that the retinal layers lacked normal segmentation and there was no evidence of a foveal depression or pit (Fig. 2e).

**Fig. 3:**
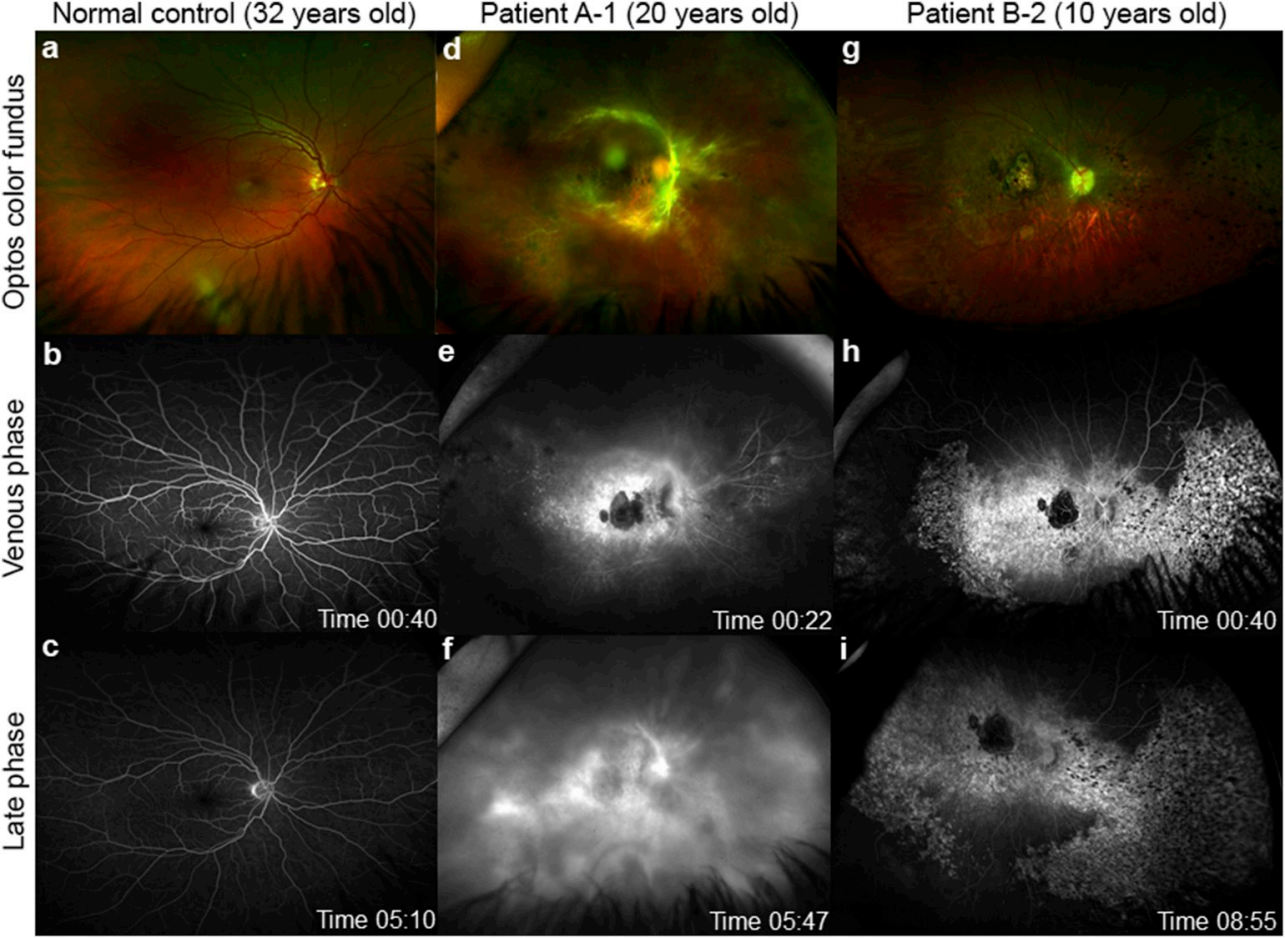
Retinal vasculature in *KCNJ13* patients. Ultra-wide field fluorescein angiography (FFA) using Optos (Dunfermline, Scotland) of the right eye from two unrelated patients with a missense mutation (p.Thr153Ile) in *KCNJ13* compared to normal control male age 32 years (a–c). Panel Patient A-1, 20 years old, (d) color fundus images showing vasoproliferative response around the arcades, widespread pan-retinal photocoagulation scars overlying the pigmentary retinopathy; (e) shows leakage (first seen in arterial phase, data not shown) in the venous phase with extensive leakage in the late phase (f). Panel Patient B-2, 10 years old, (g) color fundus images showing patchy areas of nummular pigmentary retinopathy; (h) FFA shows hyperfluorescence in areas of retinopathy with an area of hypofluorescence at the macula which coincides with delineated area of chorioretinal atrophy (choroidal vessels can be seen), but no evidence of leakage at any phase (i).

Patient A-1 developed fibrosis along the arcades and optic disc; they received extensive pan-retinal photocoagulation (PRP) and two intravitreal injections of avastin. Following a left vitreous haemorrhage, the patient had a pars plana vitrectomy with secondary complications of cataracts requiring phacoemulsification followed by uveitic glaucoma resulting in posterior capsule opacification and membranectomy. Patient A-2 also developed retinal neovascularization and right vitreous haemorrhage requiring extensive PRP. FFA was performed to investigate for ongoing retinal neovascularization and early and late leakage is seen in both patient retinas (Fig. 3d–f). FFA findings from the normal control subject were typical of a healthy retina (Fig. 3a–c) (Singer et al., 2016).

In comparison, affected siblings in family B showed characteristic features of LCA with patient B-1 showing very mild leakage from the optic disc in both eyes, but no evidence of retinal neovascularization elsewhere on FFA. Patient B-2 showed no retinovascular changes (Fig. 3g–i). However, both patients are younger than those from family A and annual monitoring is required. In the previous families diagnosed with *KCNJ1*3-LCA, where affected patients are between 10 and 34 years, no previous reports of retinovascular changes have been described (Table 1).

### 3.2 Retinal vasculature changes in the *obe*^*td15*^ zebrafish

**Fig. 4:**
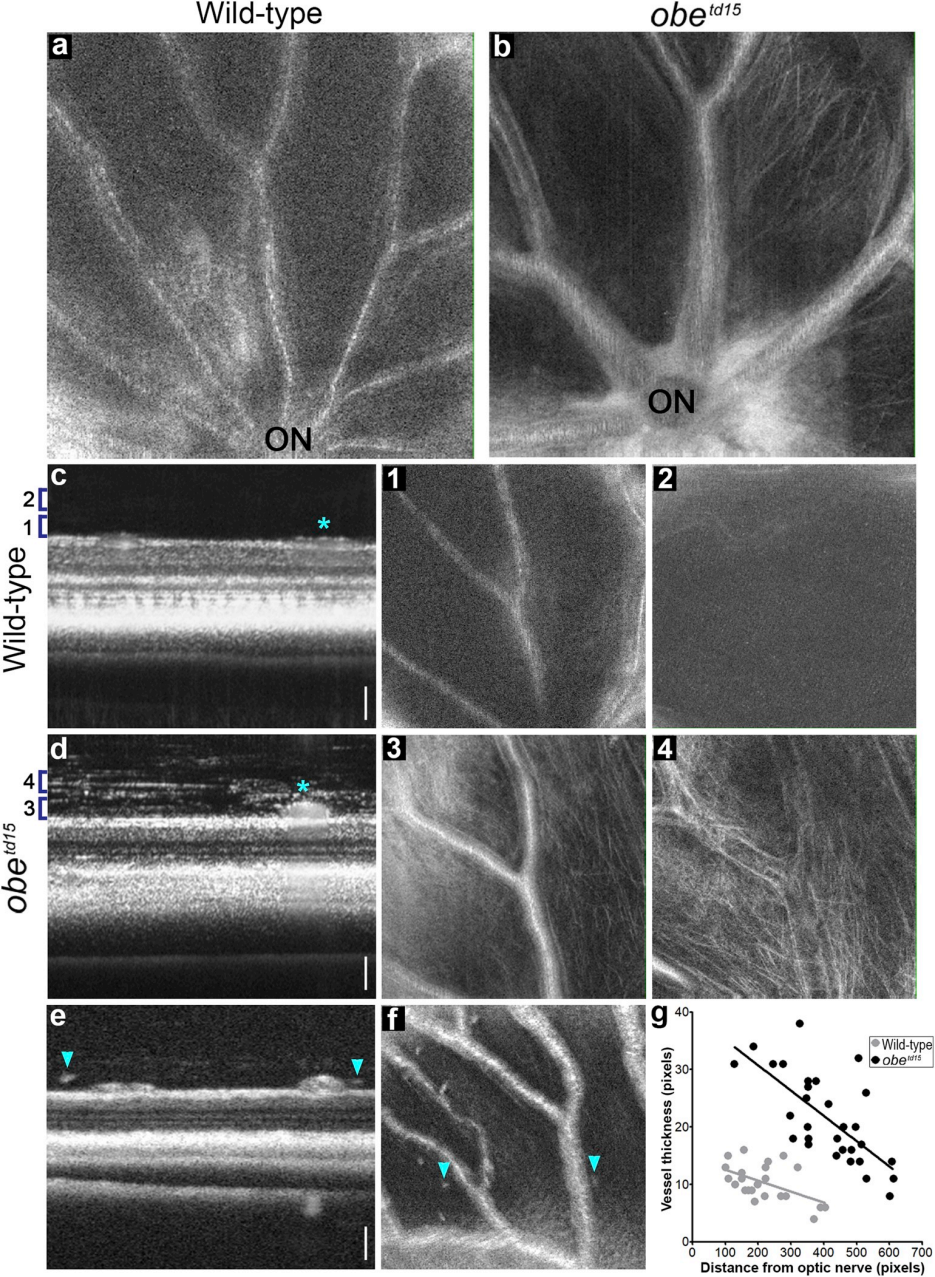
Retinal vasculature in *obe*^*td15*^ zebrafish. OCT en face images of the dorsal retina show inner retinal vessels emerging from the optic nerve region in the wild-type (a) and *obe*^*td15*^ (b) fish at 12 months post-fertilization, displaying abnormal blood vessel appearance in the mutant retina. Cross-sectional B-scans from wild-type (c) and *obe*^*td15*^ (d) fish also demonstrate notable differences in vessel size (vessels indicated with *). Numbered brackets (1–4) on the cross-sections represent areas analyzed in the corresponding en face images. Fibrous material was apparent at each depth examined in the vitreous of the *obe*^*td15*^ retina (3, 4) but was not seen on the wild-type images (1, 2). Hyper-reflective deposits were also noted in the vitreous of the *obe*^*td15*^ retina on both B-scan (e) and en face (f) images, indicated with arrows. Graph (g) shows retinal vessel thickness plotted against distance from the optic nerve in wild-type and *obe*^*td15*^ zebrafish. Scale bars = 50 μm.

The previously described *obe*^*td15*^ zebrafish showed a late onset retinal degeneration with retinal thinning and disruption of the photoreceptor and RPE layers, which became apparent at 12 mpf (Toms et al., 2019). The *obe*^*td15*^ mutation is c.502T > C, p.Phe168Leu, located within the M2 domain coding region (Fig. 1c); when the human and zebrafish Kir7.1 protein sequences are aligned, the amino acid changes carried by the *obe*^*td15*^ fish and LCA patients are separated by two residues. OCT imaging at 12 mpf revealed gross abnormalities in the appearance of the inner retinal vasculature of *obe*^*td15*^ zebrafish (Fig. 4). Comparison of age-matched wild-type and *obe*^*td15*^ central retinal vessels emerging from the optic nerve region show that the vessels appeared to be abnormally enlarged and hyper-reflective in the *obe*^*td15*^ retina compared to wild-type, allowing them to be much more easily distinguished on OCT en face projections (Fig. 4a and b). The difference in vessel thickness was also apparent on the B-scan cross-sectional images where they appear as prominent spherical structures overlying the ganglion cell layer in the *obe*^*td15*^ retina while relatively unremarkable in comparison on the wild-type images (Fig. 4c and d). Analysis of vessel width as a function of distance from the optic nerve showed a significant correlation (p < 0.001) between width and distance from the optic nerve head (Fig. 4g). Measurements were taken from 3 wild-type and 5 *obe*^*td15*^ zebrafish. The retinal vessels of *obe*^*td15*^ zebrafish were consistently wider and slopes of the trend lines were significantly different (p < 0.0001), suggesting that the mutant vessels were wider for a given distance from the optic nerve compared to wild-type controls. OCT imaging of heterozygous *obe*^*td15*^ zebrafish showed retinal vasculature comparable to that of wild-type fish (Supplementary Fig. S1).

In addition, examination of two regions at level of the vessels and just above the vessels on volume intensity projection images revealed the presence of fibrous material in the *obe*^*td15*^ vitreous which was not present in the same regions on wild-type images (Fig. 4c and d). Spot-like hyper-reflective vitreous deposits were also noted on both B-scan and en face views of the *obe*^*td15*^ retina (Fig. 4e and f).

## 4 Discussion

We have described here retinovascular abnormalities in two siblings (A-1 and A-2) with homozygous c.458C > T, p.(Thr153Ile) *KCNJ13* mutations. Both patients showed features characteristic of LCA, including chorioretinal atrophy and nummular pigmentary retinopathy. In addition, early and late leakage of retinal vessels with neovascularization and vitreous haemorrhage was observed, which have not been noted in *KCNJ1*3-LCA previously (Sergouniotis et al., 2011; Pattnaik et al., 2015; Perez-Roustit et al., 2017). These abnormalities were unlike vasoproliferative tumors, Coats-like retinopathy or the SVD phenotype. Because there is consanguinity within both of these reported families, one might suspect that perhaps another homozygous genetic variant shared between the two brothers might be responsible for their retinovascular complications. However, our findings in the zebrafish model strongly suggests that the *KCNJ13* variants alone could be responsible for this clinical feature. These novel findings within our patients and the animal model strongly suggest that this retinovascular complication should be considered within the clinical spectrum of *KCNJ13*-related retinal dystrophy (rather than as an incidental and independent finding) and perhaps dependent on the specific nature and position of the mutation and its impact on protein function.

The retinal degeneration seen in the *KCNJ13* model zebrafish, *obe*^*td15*^, has been previously described (Toms et al., 2019), showing similarity to that of LCA patients. Here, we have observed additional retinovascular abnormalities in the *obe*^*td15*^ retina where the vessels are abnormally thick and hyper-reflective on OCT images. Although there were phenotypic differences in the retinovascular changes observed in the patients and zebrafish, these may be attributed to species-related differences in vascular response; previously, adult zebrafish have shown varying responses to hypoxic (Cao et al., 2008) and hyperglycaemic (Alvarez et al., 2010) conditions, demonstrating retinal neovascularization or vessel thickening respectively, with the latter phenotype being more reminiscent of the *obe*^*td15*^ retinovascular abnormalities. The missense mutation harboured by *obe*^*td15*^ fish is located in the M2 helix coding region of the *kcnj13* gene, consistent with the location of the missense variant carried by patients A-1 and A-2, suggesting that this domain may affect vascular activity. While not identical, the amino acid changes resulting from the patient and zebrafish mutations are in very close proximity and both result in hydrophobic amino acid replacement. The M2 transmembrane domain confers the pore properties of each subunit of inwardly rectifying K^+^ channels (Wible et al., 1994).

In addition to changes in vessel structure, there were fibrous material and deposits in the vitreous of the *obe*^*td15*^ zebrafish which were not observed in our human siblings. The identity of these formations is unknown, and they may or may not be related to the vasculature. Previously, abnormal deposits have been noted on retinal examinations of *KCNJ13* patients; crystalline deposits in the retina and fibrillar degeneration of the vitreous are characteristic in patients with SVD (Lee et al., 2003), and hyper-reflective formations in the retina were described in an LCA patient, which were thought to be pigment deposits (Perez-Roustit et al., 2017). Further investigation is necessary to determine if these structures observed in the *obe*^*td15*^ zebrafish retina are related to those viewed on clinical images.

The two other younger siblings from a different family (B-1 and B-2) with the same *KCNJ13* p.(Thr153Ile) mutation did not exhibit any clinically significant retinal vascular abnormalities. This could mean that the retinovascular abnormalities might manifest itself from teenage years onwards or there could be other genes that play a role in the expression of this phenotype. For affected members of the family who have the same mutation but no vascular changes, regular monitoring would be recommended as they are not yet of the same age and the onset may be forthcoming.

In summary, we have reported novel retinovascular findings in both human LCA patients and a corresponding zebrafish mutant, associated with *KCNJ13* missense mutations. Further experimentation will be necessary to determine any involvement of Kir7.1 in vascular growth and function.

## Declaration of competing interest

None.

## References

[bib1] Alvarez Y., Chen K., Reynolds A.L., Waghorne N., O'Connor J.J., Kennedy B.N. (2010). Predominant cone photoreceptor dysfunction in a hyperglycaemic model of non-proliferative diabetic retinopathy. Dis. Model. Mech..

[bib2] Cao R., Jensen L.D., Soll I., Hauptmann G., Cao Y. (2008). Hypoxia-induced retinal angiogenesis in zebrafish as a model to study retinopathy. PLoS One.

[bib3] Doring F., Derst C., Wischmeyer E., Karschin C., Schneggenburger R., Daut J., Karschin A. (1998). The epithelial inward rectifier channel Kir7.1 displays unusual K+ permeation properties. J. Neurosci..

[bib4] Hejtmancik J.F., Jiao X., Li A., Sergeev Y.V., Ding X., Sharma A.K., Chan C.C., Medina I., Edwards A.O. (2008). Mutations in KCNJ13 cause autosomal-dominant snowflake vitreoretinal degeneration. Am. J. Hum. Genet..

[bib5] Huang Y., Cideciyan A.V., Papastergiou G.I., Banin E., Semple-Rowland S.L., Milam A.H., Jacobson S.G. (1998). Relation of optical coherence tomography to microanatomy in normal and rd chickens. Invest. Ophthalmol. Vis. Sci..

[bib6] Hughes B.A., Takahira M. (1996). Inwardly rectifying K+ currents in isolated human retinal pigment epithelial cells. Invest. Ophthalmol. Vis. Sci..

[bib7] Iwashita M., Watanabe M., Ishii M., Chen T., Johnson S.L., Kurachi Y., Okada N., Kondo S. (2006). Pigment pattern in jaguar/obelix zebrafish is caused by a Kir7.1 mutation: implications for the regulation of melanosome movement. PLoS Genet..

[bib8] Khan A.O., Bergmann C., Neuhaus C., Bolz H.J. (2015). A distinct vitreo-retinal dystrophy with early-onset cataract from recessive KCNJ13 mutations. Ophthalmic Genet..

[bib25] Krapivinsky G., Medina I., Eng L., Krapivinsky L., Yang Y., Clapham (1998). A novel inward rectifier K+ channel with unique pore properties. Neuron.

[bib9] Kumaran N., Moore A.T., Weleber R.G., Michaelides M. (2017). Leber congenital amaurosis/early-onset severe retinal dystrophy: clinical features, molecular genetics and therapeutic interventions. Br. J. Ophthalmol..

[bib10] Kusaka S., Inanobe A., Fujita A., Makino Y., Tanemoto M., Matsushita K., Tano Y., Kurachi Y. (2001). Functional Kir7.1 channels localized at the root of apical processes in rat retinal pigment epithelium. J. Physiol..

[bib11] Lee H., Deignan J.L., Dorrani N., Strom S.P., Kantarci S., Quintero-Rivera F., Das K., Toy T., Harry B., Yourshaw M., Fox M., Fogel B.L., Martinez-Agosto J.A., Wong D.A., Chang V.Y., Shieh P.B., Palmer C.G., Dipple K.M., Grody W.W., Vilain E., Nelson S.F. (2014). Clinical exome sequencing for genetic identification of rare Mendelian disorders. J. Am. Med. Assoc..

[bib12] Lee M.M., Ritter R., Hirose T., Vu C.D., Edwards A.O. (2003). Snowflake vitreoretinal degeneration: follow-up of the original family. Ophthalmology.

[bib13] Nakamura N., Suzuki Y., Sakuta H., Ookata K., Kawahara K., Hirose S. (1999). Inwardly rectifying K+ channel Kir7.1 is highly expressed in thyroid follicular cells, intestinal epithelial cells and choroid plexus epithelial cells: implication for a functional coupling with Na+,K+-ATPase. Biochem. J..

[bib26] Partiseti M., Collura V., Agnel M., Culouscou J.M., Graham D. (1998). Cloning and characterization of a novel human inwardly rectifying potassium channel predominantly expressed in small intestine. FEBS Lett..

[bib14] Pattnaik B.R., Shahi P.K., Marino M.J., Liu X., York N., Brar S., Chiang J., Pillers D.A., Traboulsi E.I. (2015). A novel KCNJ13 nonsense mutation and loss of Kir7.1 channel function causes leber congenital amaurosis (LCA16). Hum. Mutat..

[bib15] Perez-Roustit S., Marquette V., Bocquet B., Kaplan J., Perrault I., Meunier I., Hamel C.P. (2017). Leber congenital amaurosis with large retinal pigment clumps caused by compound heterozygous mutations in *KCNJ13*. Retin. Cases Brief Rep..

[bib16] Roman D., Zhong H., Yaklichkin S., Chen R., Mardon G. (2018). Conditional loss of Kcnj13 in the retinal pigment epithelium causes photoreceptor degeneration. Exp. Eye Res..

[bib17] Sergouniotis P.I., Davidson A.E., Mackay D.S., Li Z., Yang X., Plagnol V., Moore A.T., Webster A.R. (2011). Recessive mutations in KCNJ13, encoding an inwardly rectifying potassium channel subunit, cause leber congenital amaurosis. Am. J. Hum. Genet..

[bib18] Singer M., Sagong M., van Hemert J., Kuehlewein L., Bell D., Sadda S.R. (2016). Ultra-widefield imaging of the peripheral retinal vasculature in normal subjects. Ophthalmology.

[bib19] Toms M., Burgoyne T., Tracey-White D., Richardson R., Dubis A.M., Webster A.R., Futter C., Moosajee M. (2019). Phagosomal and mitochondrial alterations in RPE may contribute to KCNJ13 retinopathy. Sci. Rep..

[bib20] Toms M., Tracey-White D., Muhundhakumar D., Sprogyte L., Dubis A.M., Moosajee M. (2017). Spectral domain optical coherence tomography: an in vivo imaging protocol for assessing retinal morphology in adult zebrafish. Zebrafish.

[bib21] Wible B.A., Taglialatela M., Ficker E., Brown A.M. (1994). Gating of inwardly rectifying K+ channels localized to a single negatively charged residue. Nature.

[bib22] Yang D., Pan A., Swaminathan A., Kumar G., Hughes B.A. (2003). Expression and localization of the inwardly rectifying potassium channel Kir7.1 in native bovine retinal pigment epithelium. Invest. Ophthalmol. Vis. Sci..

[bib23] Yang D., Swaminathan A., Zhang X., Hughes B.A. (2008). Expression of Kir7.1 and a novel Kir7.1 splice variant in native human retinal pigment epithelium. Exp. Eye Res..

[bib24] Zhong H., Chen Y., Li Y., Chen R., Mardon G. (2015). CRISPR-engineered mosaicism rapidly reveals that loss of Kcnj13 function in mice mimics human disease phenotypes. Sci. Rep..

