# Supplementary material for: Missense variants in the conserved transmembrane M2 protein domain of KCNJ13 associated with retinovascular changes in humans and zebrafish
Source: Exp Eye Res. 2019 Dec;189:107852. doi: 10.1016/j.exer.2019.107852 (PMC6899441; doi:10.1016/j.exer.2019.107852)
Supplement: Multimedia component 2 [file mmc2.pdf]

Supplementary Material

Missense variants in the conserved transmembrane M2 protein domain of *KCNJ13* associated with retinovascular changes in humans and zebrafish

Maria Toms, Adam M Dubis, Wei Sing Lim, Andrew R Webster, Michael B Gorin, Mariya Moosajee

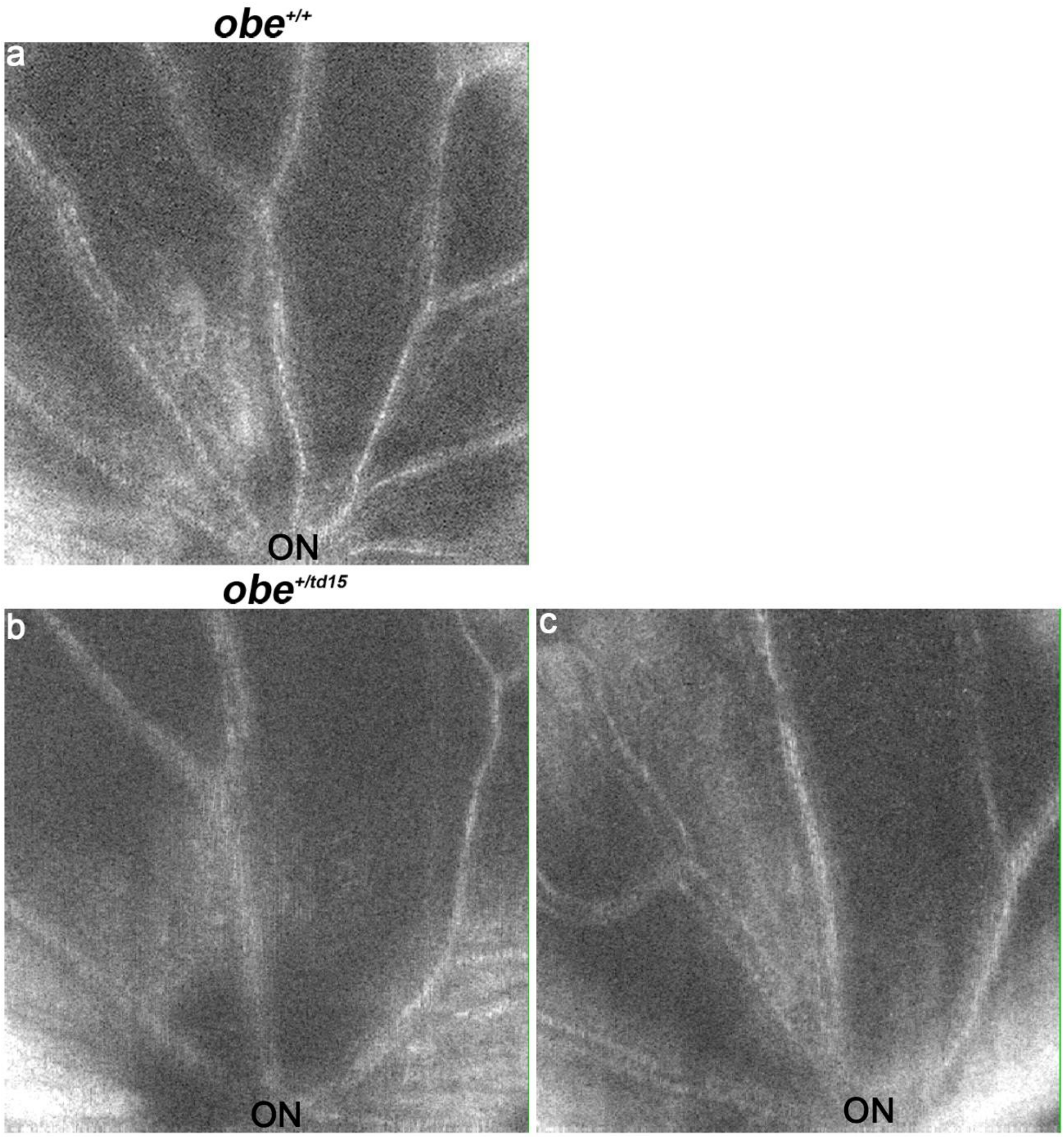

Figure S1. Retinal vasculature in heterozygous *obe*^{td15} zebrafish.
Two representative images (b, c) are shown compared to wild-type (a). ON, optic nerve.
